# Functional Magnetic Core-Shell System-Based Iron Oxide Nanoparticle Coated with Biocompatible Copolymer for Anticancer Drug Delivery

**DOI:** 10.3390/pharmaceutics11030120

**Published:** 2019-03-15

**Authors:** Thai Thanh Hoang Thi, Diem-Huong Nguyen Tran, Long Giang Bach, Hieu Vu-Quang, Duy Chinh Nguyen, Ki Dong Park, Dai Hai Nguyen

**Affiliations:** 1 Biomaterials and Nanotechnology Research Group, Faculty of Applied Sciences, Ton Duc Thang University, Ho Chi Minh City 700000, Vietnam; hoangthithaithanh@tdtu.edu.vn; 2 Institute of Applied Materials Science, Vietnam Academy of Science and Technology, Ho Chi Minh City 700000, Vietnam; trannguyendiemhuongbiotech@gmail.com; 3 NTT Hi-Tech Institute, Nguyen Tat Thanh University, Ho Chi Minh City 700000, Vietnam; vqhieu@ntt.edu.vn (H.V.-Q.); ndtrinh@ntt.edu.vn (D.C.N.); 4 Department of Chemical Engineering and Food Technology, Nguyen Tat Thanh University, Ho Chi Minh City 700000, Vietnam; 5 Department of Molecular Science and Technology, Ajou University, Suwon 443749, Korea; kdp@ajou.ac.kr; 6 Graduate University of Science and Technology, Vietnam Academy of Science and Technology, Hanoi 100000, Vietnam

**Keywords:** iron oxide nanoparticles, core-shell structure, surface modification, Poloxamer, heparin, doxorubicin

## Abstract

Polymer coating has drawn increasing attention as a leading strategy to overcome the drawbacks of superparamagnetic iron oxide nanoparticles (SPIONs) in targeted delivery of anticancer drugs. In this study, SPIONs were modified with heparin-Poloxamer (HP) shell to form a SPION@HP core-shell system for anticancer drug delivery. The obtained formulation was characterized by techniques including transmission electron microscopy (TEM), Fourier transform infrared spectra (FT-IR), vibration sample magnetometer (VSM), proton nuclear magnetic resonance (^1^H-NMR), and powder X-ray diffraction (XRD). Results showed the successful attachment of HP shell on the surface of SPION core and the inability to cause considerable effects to the crystal structure and unique magnetic nature of SPION. The core-shell system maintains the morphological features of SPIONs and the desired size range. Notably, Doxorubicin (DOX), an anticancer drug, was effectively entrapped into the polymeric shell of SPION@HP, showing a loading efficiency of 66.9 ± 2.7% and controlled release up to 120 h without any initial burst effect. Additionally, MTT assay revealed that DOX-loaded SPION@HP exerted great anticancer effect against HeLa cells and could be safely used. These results pave the way for the application of SPION@HP as an effective targeted delivery system for cancer treatment.

## 1. Introduction

Tumor-targeted drug delivery utilizing nanocarriers has emerged as a promising approach for cancer treatment [1,2,3]. Despite the great potentials in the chemotherapy of many drug delivery systems (DDS), magnetic nanoparticle (MNP), especially SPION, is the primary choice for the delivery of anticancer drugs as they are feasible to effectively target cancer cells upon the manipulation of an external magnetic field, thus improving the therapeutic efficacy of the drug [4,5,6,7]. Their superparamagnetic property allows for simultaneously treating and monitoring the tumor progression using hyperthermia therapy and magnetic resonance imaging (MRI) [8,9]. In addition, they are FDA-approved biocompatible materials and can be cleared from the body via the iron metabolic pathway [8,10]. However, the clinical applicability of SPIONs is restricted due to their physiological instability and the tendency of protein adsorption on the particles’ surface which is accountable for the inaccurate measurement of targeting effectiveness, expedited recognition of the reticuloendothelial system (RES), and in turn, the increased clearance rate of SPIONs from the bloodstream [4,11]. Moreover, drug’s conjugation to the SPIONs’ surface via covalent linkages in previous studies has been shown to give the low drug loading efficiency and the failure of the drug’s release at target region [4,8,12].

Surface engineering, where the SPION’s core is usually coated with a hydrophilic polymeric shell, has been widely investigated as a leading strategy in circumventing the disadvantages of conventional SPIONs [13]. This hydrophilic polymeric shell can (i) enhance the stability by protecting the core from oxidation; (ii) aid in the biocompatibility of SPIONs; (iii) prevent the adsorption of protein, thus reducing the entrapment of RES and prolonging the circulation time of SPIONs; and (iv) encapsulate therapeutic drugs for a better controlled release profile [4,8,14,15]. Polymers which have good biocompatibility, biodegradability, and ease of modification are more promising for nanoparticles’ modification [5,16]. Poloxamer, in particular, is a desired polymer for the stabilization of SPIONs since its surfactant effect is similar to that of oleate or poly (vinyl alcohol) (PVA), which is commonly used as stabilizing agents for magnetic nanoparticles [17,18,19]. Owing to the amphiphilic property, Poloxamer is not only capable of efficiently incorporating both hydrophilic and hydrophobic anticancer drugs, but also improving their solubility and retention time in the circulatory system [20,21,22]. Yallapu et al. developed MNPs linked with Poloxamer on the surface through β-cyclodextrin (CD) for the delivery of curcumin (CUR), a hydrophobic antitumor agent. CUR was loaded into the polymer layer via hydrophobic interaction. The CD-Poloxamer-coated MNPs have good stability with particle size varying in the range of 90.72–181.76 nm. The payload of CUR is high and proportional to the amount of CD used [23]. 

Considering the effective performance in surface engineering of SPIONs, heparin has a great potential for the modification of SPIONs to develop a better tumor-targeted delivery system for treatment of cancer [24,25]. Heparin is a hydrophilic natural polysaccharide having high biocompatibility, proper biodegradability, and low cytotoxicity [26]. It is able to improve drug bioactivity and extend the circulation time by hindering complement activation and suppressing phagocytosis [20,27,28,29]. Lately, it was proved that heparin itself can strongly interfere with the tumor development and metastasis by inducing apoptotic cell death [22]. In a study of Fazilati M et al., heparin was coated on the surface of MNPs (Hep-MNPs) for the delivery of hydrophobic cisplatin in the treatment of ovarian CP70 cancer cells. Results showed that Hep-MNPs have a mean diameter of about 45 ± 15 nm and enhanced anti-tumor effect [6]. In another study, Javid A. and co-workers decorated SPIONs with heparin, had it loaded with DOX, and investigated its effect on human cancer cell lines A2780. The system showed an average size of 110 ± 15 nm, excellent cellular uptake efficacy, and good anticancer activity [12]. 

Both Poloxamer and heparin have potential features for surface engineering of SPIONs applied in cancer treatment. Nevertheless, the overall size of these systems was remarkably increased after modification with polymers, which is much larger than the usually reported size range (6–25 nm) for MNPs to have a unique superparamagnetic property [4,8]. Besides, even though coating of polymer can increase the stability of SPIONs, the dramatical decrease in saturation magnetization may occur when using nonmagnetic materials for coating SPIONs [4,30,31]. Therefore, the optimization to develop polymer-coated SPIONs that have suitable size, improved pharmacokinetic profile, controlled release behavior, and preserved magnetic characteristics is necessary. 

In this study, a core-shell system SPION@HP, where the SPION core prepared by co-precipitation method was coated with polymeric shell of HP copolymer, was developed as a promising and effective DDS for cancer treatment. The anticancer drug of choice in this study is DOX, which was encapsulated in the HP shell of SPION@HP. The obtained formulation was characterized by various techniques including TEM, FT-IR, VSM, ^1^H-NMR, and XRD. Drug loading capacity and release behavior were also evaluated. In addition, cytotoxicity assay was performed to test the biocompatibility and anticancer activity of DOX-loaded SPION@HP.

## 2. Materials and Methods

### 2.1. Materials

Heparin sodium (150 I.U./mg min) and 4-Nitrophenyl chloroformate (NPC) were supplied by Acros Organics (Geel, Belgium). Poloxamer 407 (Mw 12,600 g/mol), iron(II) chloride tetrahydrate (FeCl_2_·4H_2_O, 99%), iron(III) chloride hexahydrate (FeCl_3_·6H_2_O, 97%), oleic acid (OA, 99%), 1-ethyl-3-[3-(dimethylamino)propyl]carbodiimide (EDC), and DOX (98–102%) were purchased from Sigma-Aldrich (St Louis, MO, USA). Tetrahydrofuran (THF) was obtained from Scharlau (Sentmenat, Spain). Diethyl ether was received from Fisher Scientific (Houston, TX, USA). All chemicals and solvents were of analytical grade and used without further purification. 

### 2.2. Synthesis of Heparin-Poloxamer (HP)

The preparation of HP copolymer underwent three steps, including the mono-activation of Poloxamer using NPC and the amination of mono-activated Poloxamer with EDA, followed by the coupling of carboxyl groups of heparin with amino groups of Poloxamer-NH_2_. Briefly, a mixture of Poloxamer (5 g) and NPC (0.18 g), in a three-necked flask, was molten and stirred at 65 °C for 5 h under nitrogen atmosphere. To solubilize the mixture, 10 mL of THF was added after the mixture was cooled out to 40 °C. The reaction was maintained overnight at room temperature. After that, distilled water (10 μL) and 3-amino-1-propanol (30 μL) was added; then the reaction mixture was stirred for another 5 h to give mono-activated Poloxamer. Next, 0.1 mL of EDA was prepared in THF for the addition of activated Poloxamer into the EDA solution. The reaction was kept at room temperature for 24 h. Poloxamer-NH_2_ was obtained when the resulting solution was precipitated in diethyl ether, filtered, and dried in vacuum overnight.

For the preparation of HP, heparin (0.1 g) was prepared in distilled water, followed by the immediate addition of 0.02 g EDC. Thereafter, Poloxamer-NH_2_ solution was added to heparin solution in a drop-wise manner. The reaction was stirred at room temperature for 24 h. The resulting solution was dialyzed against distilled water for 4 days using dialysis membrane (MWCO 50 kDa, Spectrum Laboratories, Inc., Rancho Dominguez, CA, USA) before freeze-drying to obtain HP in powder form.

### 2.3. Preparation of SPIONs and SPION@HP Core-Shell System

SPIONs were prepared by co-precipitation method as previously reported with modification [32]. Initially, 80 mL mixture of FeCl_3_·6H_2_O (0.2 M) and FeCl_2_·4H_2_O (0.1 M) (molar ratio of Fe^2+^:Fe^3+^ = 1:2) was added to the three-neck flask and constantly stirred under nitrogen atmosphere. Next, the pH of the mixture was adjusted by NH_4_OH solution (10%, w/w). The reaction was maintained at room temperature for 1 h until the pH reached 10, changing the color of the solution to dark black. After that, the precipitate was isolated from the solution by using a super magnet bar and then washed with deionized water at least three times.

To obtain SPION@HP, SPIONs (154 mg) dispersed in 50 mL of deionized water was added to the HP solution containing 300 mg of HP dissolved in 50 mL of deionized water. The mixture was maintained at room temperature for 6 h under ultra-sonication. During this process, HP copolymer was adsorbed on the surface of SPIONs. The obtained product was centrifuged at 14,000 rpm and lyophilized (freezing temperature −40 °C) for further use.

### 2.4. Characterizations

The chemical structure of HP copolymer was analyzed by ^1^H-NMR (NMR-400, Varian, CA, USA) using deuterium oxide (D_2_O) as a solvent. FT-IR analysis of bare SPIONs, HP, and SPION@HP was carried out using FT-IR spectrophotometer (Frontier MIR/FIR, Perkin Elmer, Waltham, MA, USA) acquired in the range of 500–4000 cm^−1^ with a resolution of 4 cm^−1^. Samples were prepared by mixing with KBr and pressed into a pellet. Size and morphology of the obtained core-shell system after polymeric coating as compared to un-modified SPIONs were illustrated by TEM (JEM-1400, Tokyo, Japan). Crystalline structure and the corresponding sizes of SPIONs and SPION@HP were analyzed by Rigaku D/Max-2550 V diffractometer at a scanning rate of 4°/min in the 2θ range of 30–70° (λ = 0.15405 nm, 40 kV, 40 mA). The magnetization curves of the products were investigated using a vibrating sample magnetometer (EV11, Lowell, MA, USA) at room temperature from −15 to 15 kOe.

### 2.5. Drug Loading and In Vitro DOX Release Study

For the preparation of DOX-loaded SPION@HP, 10 mg of DOX and 100 mg of SPION@HP were mixed together in 10 mL of deionized water. The mixture was sonicated for 60 min, kept under slow stirring for 24 h, and then dialyzed against deionized water for 8 h at room temperature to remove the free drug from the formulation using a dialysis membrane (MWCO 3.5 kDa). The deionized water was changed 5–6 times per day. The product was lyophilized to obtain DOX-loaded SPION@HP. The drug loading efficiency (DLE) and drug loading content (DLC) were quantified by UV-Vis spectrophotometer (NIR-V670-JASCO, Easton, MD, USA) using the following equations [8]:

(1) $$\mathrm{DLE}\;\left(\text{\%}\right)=\frac{\mathrm{weight}\;\mathrm{of}\;\mathrm{fed}\;\mathrm{drug}\;-\;\mathrm{weight}\;\mathrm{of}\;\mathrm{unloaded}\;\mathrm{drug}}{\mathrm{weight}\;\mathrm{of}\;\mathrm{fed}\;\mathrm{drug}}\;\times \;100$$

(2) $$\mathrm{DLC}\;\left(\text{\%}\right)=\;\frac{\mathrm{weight}\;\mathrm{of}\;\mathrm{fed}\;\mathrm{drug}\;-\;\mathrm{weight}\;\mathrm{of}\;\mathrm{unloaded}\;\mathrm{drug}}{\mathrm{weight}\;\mathrm{of}\;\mathrm{drug}\;\mathrm{loaded}\;\mathrm{SPION}\text{@}\mathrm{HP}\;}\;\times \;100$$

For the in vitro release study, 1 mL of DOX-loaded SPION@HP core-shell system suspended in PBS (DOX content, 0.3 mg/mL) was transferred to a dialysis bag (MWCO 12–14 kDa) and immersed into the vial containing 14 mL of 0.01 M phosphate buffer. The vials were horizontally shaken at 100 rpm and maintained at 37 °C in an orbital shaker bath. The release medium (14 mL) was collected, filtered (pore size = 0.20 μm), lyophilized at specific time intervals (1, 3, 6, 12, 24, 36, 48, 72, 96, and 120 h), and replaced with an equal volume of fresh media. The amounts of released DOX were determined in triplicate by UV-Vis spectrophotometer.

### 2.6. Cytotoxicity Study

The cytotoxicity of the obtained products was determined by standard MTT assay as described previously [8]. To be specific, the culture of Hela cells was carried out in 130 μL of DMEM (Dulbecco’s Modified Eagle’s medium) supplemented with 10% fetal bovine serum and 1% penicillin-streptomycin. Following that, a 96-well disc was seeded at a density of 1 × 10^4^ cells/well with a culture duration of 24 h under a humidified environment. The environment in which the disc is maintained contained 5% CO_2_, at 37 °C. The media were then discarded and replaced with fresh media containing samples alone (HP, SPIONs, and SPION@HP), free DOX, or DOX-loaded core-shell system. In control wells, cells were treated with medium only and assigned to 100% survival. Wells containing medium only but no cells served as a background control (blank). After 48 h of incubation, the supernatant from each well was removed, followed by two cell washes with PBS. Next, MTT solution (25 μL, 2 mg/mL in PBS) and culture medium (130 μL) were added. The cells were further incubated for 3 h, followed by the removal of media with constant MTT. To dissolve the purple formazan precipitate, DMSO (130 μL) was added. The absorbance was recorded at 570 nm using a multi-plate reader (SpectraMax M2e, Molecular Devices Co., Sunnyvale, CA, USA). The relative cell viability was calculated by normalizing the absorbance intensity of samples to that of the control group with the following equation:

(3) $$\mathrm{Cell}\;\mathrm{viability}\;\left(\text{\%}\right)=\frac{\left({\left[\mathrm{Abs}\right]}_{\mathrm{sample}}-\;{\left[\mathrm{Abs}\right]}_{\mathrm{blank}}\right)}{\left({\left[\mathrm{Abs}\right]}_{\mathrm{control}}-\;{\left[\mathrm{Abs}\right]}_{\mathrm{blank}}\right)}\;\times \;100$$

### 2.7. Statistical Analysis

Quantitative data were expressed as mean ± standard deviation for *n* = 3. The statistical analysis was performed by using ANOVA followed by Student’s *t*-test with *p* < 0.05 considered statistically significant.

## 3. Results and Discussion

### 3.1. Characterizations of SPION@HP

**Figure 1:**
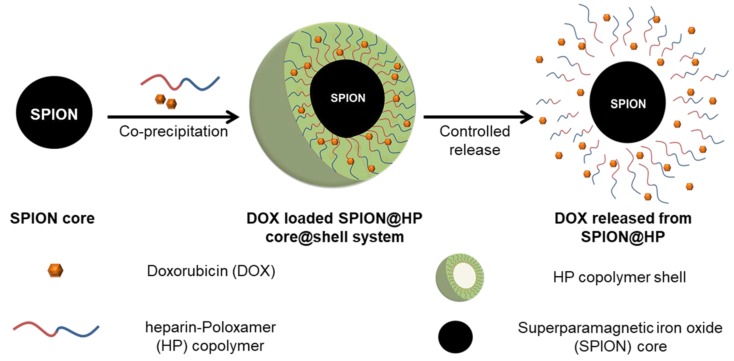
Illustration showing the coating HP copolymer onto SPION, loading of DOX to SPION@HP for the formation of DOX-loaded SPION@HP core@shell system, and the sustained release at a steady rate of DOX by diffusing through the polymeric matrix as the HP shell degraded.

**Figure 2:**
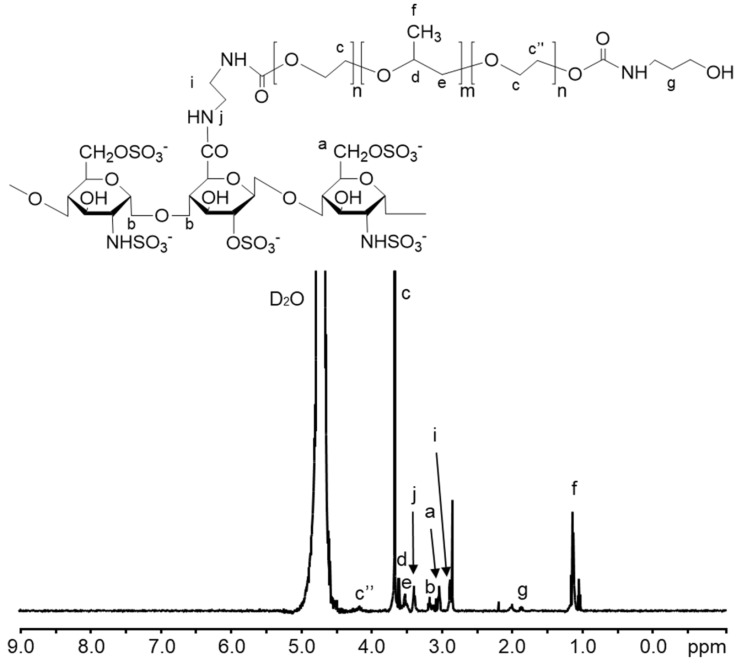
^1^H-NMR spectrum (D_2_O, δ in ppm) of the HP copolymer and chemical structure of HP. Signals (a-g) were assigned to HP’s structure.

**Figure 3:**
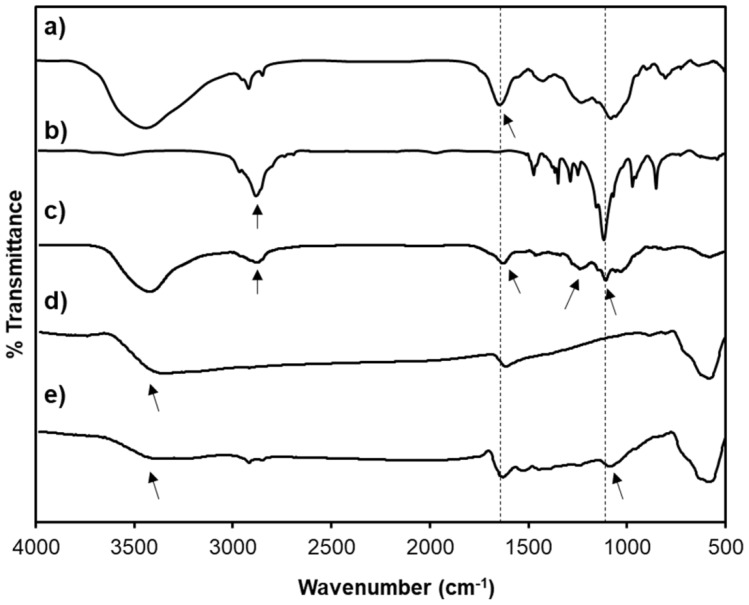
FT-IR spectra of heparin (**a**); Poloxamer (**b**); HP (**c**); SPIONs (**d**); and SPION@HP (**e**).

The fabrication of the SPION@HP core-shell system was performed by decorating HP polymeric shell onto the surface of SPION core prepared by the co-precipitation method (Figure 1). Herein, heparin-Poloxamer is a graft copolymer comprising of a backbone of heparin polymer to which a number of Poloxamer sequences are randomly grafted onto heparin via the coupling of carboxyl groups of heparin and amino groups of Poloxamer-NH_2_. ^1^H-NMR spectroscopy was employed to characterize the structure of the synthesized HP copolymer. Figure 2 indicated that the ^1^H-NMR spectrum of the HP conjugate has peaks at 3.80–3.55 and 1.24 ppm that were attributed to polyethylene oxide and polypropylene oxide blocks of Poloxamer. The signal at 4.15 ppm referred to the conjugation of the amino propanol terminal. Besides, the presence of EDA’s methylene proton signal at 2.98 ppm together with the complete absence of NPC’s aromatic protons at 7.40 and 8.27 ppm and methylene proton at the terminal of polyethylene oxide block indicate that all NPC moieties were substituted by EDA. Moreover, a new signal at 3.4 ppm was assigned to NH-CO, confirming the conjugation of HP. Additionally, FT-IR was carried out to further confirm the structure of the synthesized products. Chemical structures of (a) heparin, (b) Poloxamer, (c) HP, (d) SPIONs, and (e) SPION@HP are demonstrated in Figure 3. As shown in the spectrum of HP (Figure 3c), typical bands of C-O-C and –SO_2_-O- at 1110 and 1245 cm^−1^ indicate the presence of both Poloxamer and heparin, respectively, in the conjugate. The signal at 2900–3000 cm^−1^ of C-H stretching was observed only in the spectra of HP and Poloxamer but was absent in the spectrum of heparin. A shift in the characteristic band of C=O stretching vibration from 1680 cm^−1^ in the heparin spectrum to 1610 cm^−1^ was observed, indicating the amide formation between heparin and Poloxamer-NH_2_. In Figure 3d,e, it is clear that typical bands corresponding to the Fe-O/Fe-O-Fe bonds of SPIONs could be observed in the spectrum of both SPIONs and SPION@HP at 571 cm^−1^ and 578 cm^−1^, respectively [7]. The presence of OH groups on the surface of SPIONs was also identified by bands at 3416 cm^−1^ (Figure 3d) and 3420 cm^−1^ (Figure 3e). For SPION@HP, the OH stretching vibration of HP at around 3430 cm^−1^, which was overlapped by the band of surface OH groups of SPION, was observed. Distinctive bands of heparin and Poloxamer were also evidently detected. In comparison to SPION’s spectrum, a strong shift of Fe-O stretching from 571 to 578 cm^−1^ in SPION@HP spectrum could be explained by the coating of the HP shell. Besides, the C-O stretching band of copolymer at 1110 cm^−1^ slightly shifted to a lower wavelength (1090 cm^−1^) due to the hydrogen bonds and electrostatic forces created between the magnetic core and the polymer shell, which is consistent with a previous study [7]. These results proved that HP was successfully attached to the surface of SPIONs.

**Figure 4:**
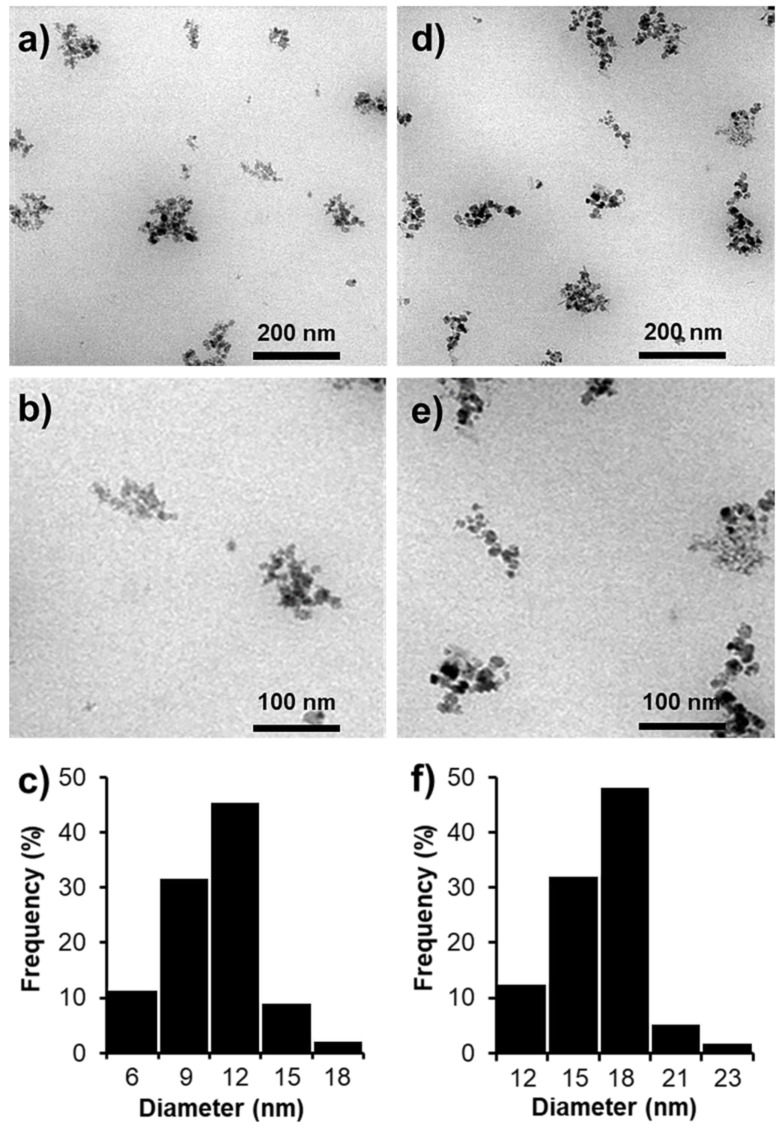
TEM images (**a**,**b**,**d**,**e**) and particle size distributions (**c**,**f**) of SPIONs (**a**–**c**) and SPION@HP (**d**–**f**), respectively (scale bar = 200 nm and 100 nm).

The size and morphology of SPIONs and SPION@HP were determined by TEM. Figure 4 shows that the average diameters of SPIONs and SPION@HP were 11.6 nm and 17.7 nm, respectively. As compared to SPIONs, SPION@HP (Figure 4b) still retains the morphological features of SPIONs except for the increase in the particle size, implying the uniform covering of HP shell outside of the SPION’s core. This polymeric shell is expected to protect the magnetic core from the oxidation in physiological condition, thus improving the stability of SPIONs. The hydrophilic nature of the shell can also prevent the adsorption of protein on the surface of the delivery system through steric repulsion and then escape the capture of RES and extend their fate in the circulatory system [33,34]. More importantly, we observed that the increase in size of SPION@HP is modest, making their size still in the desired range (6–25 nm) in which a MNP could gain its unique superparamagnetic properties [4,30,31].

**Figure 5:**
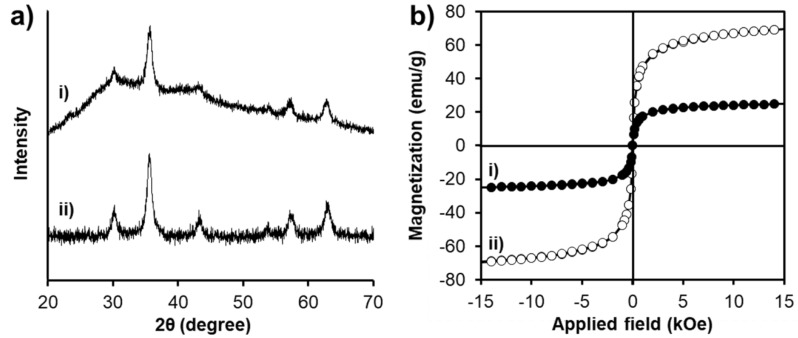
XRD patterns (**a**) and magnetization curves (**b**) of SPION@HP (i) and SPIONs (ii).

XRD patterns of SPION@HP (Figure 5(a-i)) and SPIONs (Figure 5(a-ii)) are investigated to determine whether the surface modification with HP polymeric shell could affect the structure of the SPION’s core. Similar to the XRD pattern of SPIONs, the characteristic peaks marked by their corresponding indices (2 2 0), (3 1 1), (4 0 0), (4 2 2), (5 1 1), and (4 4 0), also presented in the XRD pattern of SPION@HP. In fact, these six diffraction peaks, indicating cubic iron oxide crystals (2θ = 30.08°, 35.38°, 42.84°, 53.34°, 56.92°, and 62.42°), are consistent with the Joint Committee of Powder Diffraction Standards (JCPDS) database for magnetite (card No. 89-3854) [35]. This affirms that the crystal structure of SPIONs is preserved after polymeric coating. Besides, XRD measurement revealed that the crystallite size D (3 1 1) of SPIONs and SPION@HP were 2.53 and 2.52 Å, whilst the particle sizes were 12 and 18 nm, which were calculated by the Debye–Sherrer method. This particle size result is nearly the same with the result obtained by TEM.

The magnetization curves of the core-shell structure are depicted in Figure 5b, in which the saturation magnetization (Ms) value of SPION@HP was 24.92 emu g^−1^ while that of SPIONs was 68.9 emu g^−1^. It is apparent that a sharp decline in the Ms occurred after the coating of non-magnetic polymeric materials to the SPIONs’ surface. Nonetheless, the Ms value of SPION@HP is sufficiently high for most of the clinical and biomedical applications including the drug delivery [30,36]. Furthermore, SPION@HP could be separated quickly and easily from the solution when using the magnet, thus facilitating the delivery of drug at the target site upon the magnetic field treatment.

### 3.2. Loading Efficiency and Controlled Release of DOX

**Figure 6:**
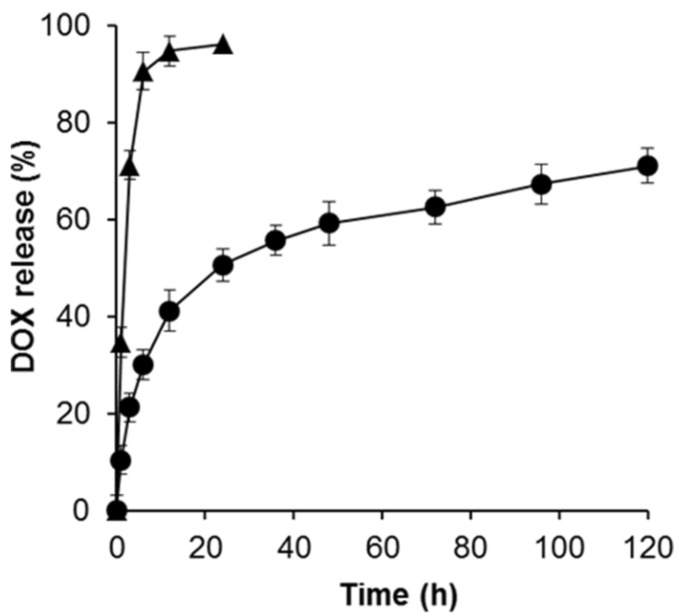
Cumulative release profile of DOX from SPION@HP (●) at predetermined time intervals. Each marked point corresponds to 0, 1, 3, 6, 12, 24, 36, 48, 72, 96, and 120 h. The release of free DOX (▲) was carried out as a control, in which the amount of free DOX was equivalent to the amount of loaded DOX in the SPION@HP. All experiments were performed three times in PBS (0.01 M) at 37 °C.

Regarding DDSs, drug loading efficiency is a critical parameter that directly affects the therapeutic efficacy of the system. Herein, DOX was successfully encapsulated into the HP shell of the SPION@HP. The DLE was found to be 66.9 ± 2.7%, indicating the relatively high loading capacity of SPION@HP. In addition to loading capacity, the release behavior of DOX-loaded SPION@HP was also elucidated since the capability of a DDS for temporally controlled release of loaded cargo is required to improve the therapeutic effectiveness and reduce the side effects of anticancer drugs [8]. The release of free DOX with an equivalent amount to that loaded in SPION@HP was also carried out as a control under the same experimental condition. Being different from other studies that directly conjugate a drug or chemically-modified drug to SPIONs’ surface via strong covalent linkage, leading to the failure of the drug’s release at a target region [4,12,17], DOX was effectively entrapped into the polymeric matrix of the HP shell without using any chemical conjugation. This benefits the delivery of DOX by preserving its native biological activities and allowing for the sustained and controlled release of the drug from the core-shell system. As shown in Figure 6, only 21.4% of DOX was released from SPION@HP while 71.2% of free DOX was released during the first 3 h. Within 1 day, free DOX was released almost completely (96.2%) whereas the cumulative release amount of DOX from SPION@HP only reached 50.7%. This drug release behavior is consistent with the results of previous studies where MNPs were coated with PLGA or Pluronic [17,36]. The sustained release at a steady rate of DOX-loaded SPION@HP continuously lasted up to 120 h without an initial burst release, thus maintaining the therapeutic level of therapeutics in a longer time for the treatment. This could be explained by the slow diffusion of the drug through the polymeric matrix as the HP shell degraded. Taken together, SPION@HP might serve as a high potential candidate for controlled DDS.

### 3.3. Cytotoxicity Study

**Figure 7:**
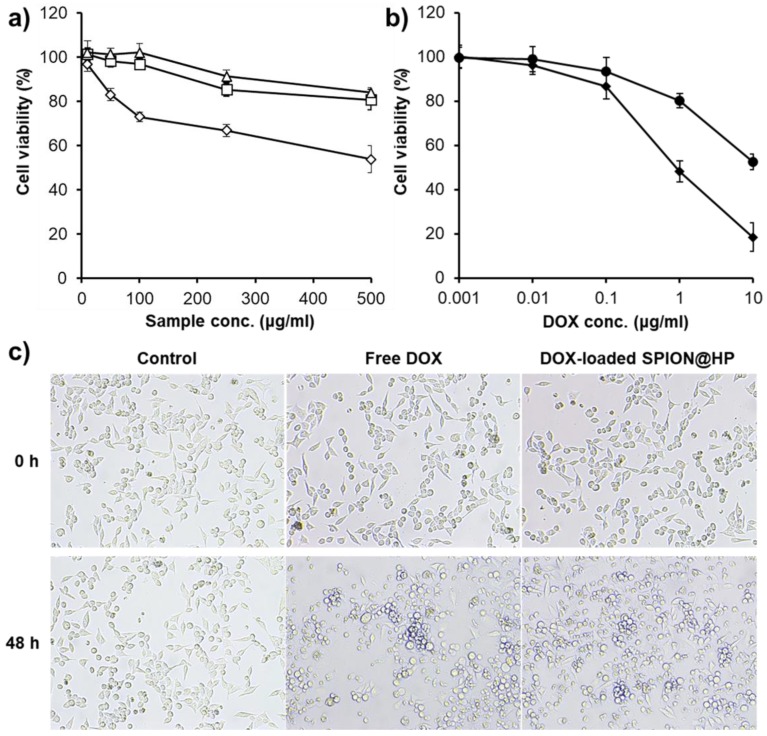
(**a**) Viability of HeLa cells when treated with HP (△), SPIONs (◇), and SPION@HP (◻); (**b**) dose-dependent cytotoxicity of DOX-loaded SPION@HP (●) and free DOX (◆) after 48 h of incubation; and (**c**) image of HeLa cells incubated with free DOX and DOX-loaded SPION@HP at 10 μg mL^−1^. Data are expressed as mean ± SD (*n* = 3).

The in vitro cytotoxicity of the materials, free DOX, and DOX-loaded SPION@HP against HeLa cells were investigated using MTT assay. Figure 7a shows that both HP and bare SPION@HP, even at the highest concentration (500 μg mL^−1^), exhibited no cytotoxicity since more than 80% of cells still survived after 2 days of incubation. In contrast, nearly half of the cells died at the equivalent concentration of SPIONs. It could be observed that SPION@HP is more cytocompatible than un-modified SPIONs thanks to the coating of a highly biocompatible HP shell onto the surface of SPIONs. Besides, the concentration-dependent cytotoxicity is reported in Figure 7b when treating cells with increasing doses of free DOX and loaded DOX in SPION@HP. Approximately 48% of cancer cells were killed after being treated with 10 μg mL^−1^ of DOX-loaded SPION@HP whereas up to 82% of dead cells were observed with free DOX at the same concentration. The more dramatic decrease in cell viability at the equivalent amount of free DOX could be explained by the instant exposure of DOX to the cells, which was reported in previous literatures [37]. By well encapsulating the DOX inside the HP shell during the delivery process in the bloodstream and sustaining the release of encapsulated DOX at the target site, DOX-loaded SPION@HP is expected to have low systemic toxicity for controlled delivery of an anticancer drug in cancer treatment.

## 4. Conclusions

SPION@HP was successfully prepared and characterized for the delivery of DOX. This core-shell system had the desired size range and relatively high drug loading efficiency without significant changes in their morphological features, crystal structure, and unique magnetic property as compared with un-modified SPIONs. The controlled release of DOX from SPION@HP was successful in vitro up to 120 h without any initial burst effect. Moreover, DOX-loaded SPION@HP exerted a great anticancer effect against HeLa cells and could be safely used. Given these obtained results, the SPION@HP core-shell system should be proposed as a promising DDS for the treatment of cancer, utilizing the synergistic effect of chemotherapy and hyperthermia.
